# Altered Peripheral Blood Monocyte Phenotype and Function in Chronic Liver Disease: Implications for Hepatic Recruitment and Systemic Inflammation

**DOI:** 10.1371/journal.pone.0157771

**Published:** 2016-06-16

**Authors:** Victoria L. Gadd, Preya J. Patel, Sara Jose, Leigh Horsfall, Elizabeth E. Powell, Katharine M. Irvine

**Affiliations:** 1 Centre for Liver Disease Research, School of Medicine, The University of Queensland, Translational Research Institute, Brisbane, Australia; 2 Department of Gastroenterology and Hepatology, Princess Alexandra Hospital, Brisbane, Australia; Centre d'Immunologie et des Maladies Infectieuses,INSERM, FRANCE

## Abstract

**Background and Aims:**

Liver and systemic inflammatory factors influence monocyte phenotype and function, which has implications for hepatic recruitment and subsequent inflammatory and fibrogenic responses, as well as host defence.

**Methods:**

Peripheral blood monocyte surface marker (CD14, CD16, CD163, CSF1R, CCR2, CCR4, CCR5, CXCR3, CXCR4, CX3CR1, HLA-DR, CD62L, SIGLEC-1) expression and capacity for phagocytosis, oxidative burst and LPS-stimulated TNF production were assessed in patients with hepatitis C (HCV) (n = 39) or non-alcoholic fatty liver disease (NAFLD) (n = 34) (classified as non-advanced disease, compensated cirrhosis and decompensated cirrhosis) and healthy controls (n = 11) by flow cytometry.

**Results:**

The selected markers exhibited similar monocyte-subset-specific expression patterns between patients and controls. Monocyte phenotypic signatures differed between NAFLD and HCV patients, with an increased proportion of CD16^+^ non-classical monocytes in NAFLD, but increased expression of CXCR3 and CXCR4 in HCV. In both cohorts, monocyte CCR2 expression was reduced and CCR4 elevated over controls. CD62L expression was specifically elevated in patients with decompensated cirrhosis and positively correlated with the model-for-end-stage-liver-disease score. Functionally, monocytes from patients with decompensated cirrhosis had equal phagocytic capacity, but displayed features of dysfunction, characterised by lower HLA-DR expression and blunted oxidative responses. Lower monocyte TNF production in response to LPS stimulation correlated with time to death in 7 (46%) of the decompensated patients who died within 8 months of recruitment.

**Conclusions:**

Chronic HCV and NAFLD differentially affect circulating monocyte phenotype, suggesting specific injury-induced signals may contribute to hepatic monocyte recruitment and systemic activation state. Monocyte function, however, was similarly impaired in patients with both HCV and NAFLD, particularly in advanced disease, which likely contributes to the increased susceptibility to infection in these patients.

## Introduction

Monocytes are heterogeneous and highly plastic cells that play critical roles in host defence and tissue homeostasis. Experimental models demonstrate that peripheral blood monocytes continuously traffic to (and probably from [1]) the healthy liver, but are recruited in increased numbers in the setting of liver injury, driving liver inflammation and fibrogenesis [2–5]. We and others have previously reported elevated numbers of liver monocytes/macrophages from the early stages of chronic liver disease (CLD) in patients with chronic hepatitis C (HCV) and non-alcoholic fatty liver disease (NAFLD)[6–8], in the absence of evidence of local proliferation, supporting a role for infiltrating monocyte-derived macrophages in human disease progression.

Human monocytes are broadly classified into three phenotypically and functionally distinct subsets, based on CD14 and CD16 expression; which likely represent different stages of maturity and differentiation [9]. ‘Classical’ CD14^high^/CD16^-^ monocytes (comprising ~80% of peripheral blood monocytes) express high levels of chemokine (C-C motif) receptor (CCR)2 and exhibit strong phagocytic capacity. CD16^+^ monocytes, which preferentially express the chemokine (C-X3-C motif) receptor (CX3CR)1, were traditionally designated pro-inflammatory, although recent evidence supports a prominent role for the CD14^high^CD16^+^ ‘intermediate’ subset in inflammation, and angiogenic and surveillance functions for the CD14^+^/CD16^+^ ‘non-classical’ subset [9, 10]. Alterations in monocyte subsets, in particular an increase in intermediate and/or non-classical monocytes, are frequently observed in infectious and inflammatory diseases, and are associated with clinical outcomes [10–12]. However the relationship between circulating monocytes and innate immune-driven disease processes at the site of injury is complex and context dependent.

Multiple chemokines are reported to be elevated in the liver and serum of patients with CLD [13, 14] and regional differences in the expression of intrahepatic chemoattractants [15, 16] may be responsible for regional localisation of distinct leukocyte populations [6, 7, 15]. Although a key role for classical monocytes and the CCR2/chemokine (C-C motif) ligand (CCL)2 axis in driving liver inflammation and fibrogenesis has been demonstrated in mice [2, 4, 5], preferential accumulation of hepatic and, in some studies peripheral, CD16^+^ monocytes has been reported in human CLD [8, 11, 17, 18], especially in areas of active inflammation and fibrosis [19]. Evidence suggests that both enhanced recruitment of CD16^+^ monocytes and local differentiation from CD16^-^ precursors contribute to the preferential accumulation of CD16^+^ monocytes in the liver [8, 19]. Whether peripheral blood monocyte subsets are altered in patients with CLD of different etiologies or at different stages of disease, and how they are recruited and contribute to disease progression, is not well understood.

In addition to supplying the liver with macrophage and dendritic cell precursors, circulating monocytes are functional innate immune cells, mediating host defence against microbial pathogens through phagocytosis, production of reactive oxygen species (ROS) and inflammatory and regulatory cytokines. Innate immune dysfunction likely contributes to the high susceptibility to infection in patients with advanced liver disease [20, 21]. Increased numbers of immunosuppressive monocytes were observed in patients with acute on chronic liver failure [22], and reduced monocyte major histocompatibility complex class II surface receptor (HLA-DR)(involved in antigen presentation and T-cell activation) levels predicted adverse prognosis and paralleled disease severity in critically ill patients with cirrhosis [23]. Poor peritoneal monocyte phagocytic and bactericidal capacity was associated with infection in patients with cirrhosis and ascites [21], but whether circulating monocyte function is impaired in people with CLD, and at what stage of disease, has not been widely studied.

The first aim of this study was to characterise the phenotype of circulating monocytes in patients with chronic HCV infection and NAFLD. We sought to determine whether alterations in monocyte subsets or chemokine receptor expression are seen in patients with different etiologies or stages of liver disease, in order to gain insight into the recruitment signals responsible for hepatic monocyte/macrophage accumulation. The second aim was to assess the function of circulating monocytes, to determine whether monocyte immunocompetence is impaired with CLD progression.

## Materials and Methods

### Patients and clinical data

Non-fasting peripheral blood samples (12 mL) were collected from inpatients or people reviewed in the hepatology outpatient clinic at the Princess Alexandra Hospital, Brisbane, Australia. Demographic and clinical characteristics, including complete blood counts and blood chemistry, and survival data were obtained from research nurse interview and medical records. Diagnosis of liver disease was based on standard biochemical and serological assays. Chronic HCV infection was confirmed by detection of circulating HCV RNA. A diagnosis of NAFLD required demonstration of hepatic steatosis by imaging or liver biopsy in the presence of metabolic risk factors and the exclusion of significant alcohol consumption (<10g/day for women and <20g/day for men) and other causes of hepatic steatosis or other chronic liver disease. Liver disease severity was broadly classified by a hepatologist (EEP) as “no advanced disease”, “advanced fibrosis” (determined by transient elastography, liver biopsy if available or liver imaging consistent with cirrhosis and/or portal hypertension), or “decompensated liver disease” (determined by the presence of ascites, hepatic encephalopathy, variceal bleeding or jaundice). Patients with non-advanced fibrosis, compensated and decompensated cirrhosis had median fibroscan readings of 6.3, 14.6 and 43, respectively (data available for 49 patients). Patients with compensated and decompensated cirrhosis had median model for end stage liver disease (MELD) scores of 8 and 15.5 respectively. Eleven blood samples collected from healthy volunteers matched by age and sex were used as control samples to obtain non-diseased values. The protocol was approved by The University of Queensland and Metro South Hospital and Health Services Human Research Ethics Committees, and written informed consent was obtained from each patient and volunteer.

### Phenotypic assessment of peripheral blood mononuclear cells

Peripheral blood mononuclear cells (PBMC) were isolated using Ficoll-PAQUE (GE Healthcare Life Sciences) as described in the manufacturer’s manual before cryopreservation. Phenotyping panels comprised conventional monocyte subset and activation markers and candidate receptors relevant to liver recruitment, identified by literature search and reanalysis of public microarray data (GSE33650 [24], GSE40184 [25]). 0.5–1x10^6^ cells were resuspended in FACS Buffer (PBS/2% FBS/5 mM EDTA) and stained with optimised panels comprising the following antibodies CD14-BV421 (M5E2), CD163-PerCP-Cy5.5 (GHI/61), CSF1R-PE (AFS98), CCR4-PerCP-Cy5.5 (L291H4), CCR5-PE (2D7), HLA-DR-FITC (L243), CXCR3-PE-Cy7 (G025H7), CXCR4-PerCP-Cy5.5 (12G5), CD62L-FITC (DREG-56), SIGLEC-1-APC (7–239) (all BioLegend), CD16-APC-H7 (3G8; BD Biosciences) and CCR2-APC (48607; R&D Systems) on ice for 30 minutes in the dark. Each panel included CD14 and CD16 to identify the 3 main monocyte subsets as well as LIVE/DEAD Aqua (Molecular Probes) to label non-viable cells. The median fluorescence intensity (MFI) of each monocyte subset marker was normalized by subtracting the MFI of the corresponding fluorescence-minus-one control channel (S1 Fig). Unstained, single colour and fluorescence-minus-one control tubes were performed to generate compensation matrices that correct for spectral overlap and assist with gating.

### Ex vivo functional assays

Monocyte function was assessed in EDTA-coagulated whole blood within 2 hours of collection. Monocyte phagocytic capacity was assessed by the uptake of pHrodo^™^ *E*.*coli* BioParticles^®^ (Molecular Probes), which fluoresce in the decreased pH environment of phagosomes. 100μL of whole blood was incubated with 10μL of 1mg/mL (~3x10^6^ BioParticles) stock for 60 minutes at 37°C in the presence of monocyte surface marker antibodies, followed by red blood cell lysis (0.17M NH4Cl/0.001M EDTA/0.01M Tris, pH 7.4) and 2 washes in PBS.

Dichlorodihydrofluorescein diacetate (DCFH-DA; Molecular Probes) was used to assess monocyte ROS. 100μL of whole blood was loaded with 50μM DCFH-DA, together with monocyte surface marker antibodies for 15 minutes at 37°C. The samples were stimulated for 15 minutes with 200 μg/mL zymosan opsonised with normal human serum (30 minutes at 37°C), or left unstimulated, followed by red blood cell lysis.

To assess monocyte-specific tumour necrosis factor (TNF) production, 200μL of whole blood was stimulated with lipopolysaccharide (LPS) (100ng/mL), or left unstimulated, in the presence of the protein transport inhibitor Brefeldin A (10μg/mL; Sigma) for 4 hours at 37°C, with the addition of monocyte surface marker antibodies 45 minutes before the end of culture. Following red blood cell lysis samples were fixed in 4% paraformaldehyde for 5 minutes, washed in permeabilisation buffer (FACS Buffer containing 0.5% saponin) and stained for intracellular TNF-APC (MAb11; BioLegend).

All phenotypic and functional assay samples were washed twice in PBS and resuspended in 1% paraformaldehyde for analysis, acquired using Beckman Coulter Gallios flow cytometer and analysed using Kaluza Analysis Software.

### Statistical Analysis

MFI values were log transformed for statistical analysis to normally distribute the data. Statistical analyses utilised one-way analysis of variance, with Tukey’s or Dunnett’s post-test, the Student’s t test and Spearman’s rank-correlation coefficient. A P value of <0.05 was defined as the level of significance and all graphs and analyses were performed using Graph Pad Prism (version 6.04; GraphPad Software Inc.).

## Results

### Patient characteristics at venesection

Of a total of 73 patients, 39 patients had chronic HCV and 34 had NAFLD. A cohort of 11 age and sex-matched healthy volunteers was included to obtain non-diseased values. Demographic and clinical characteristics of the subjects at the time of venesection are summarised in Table 1.

**Table 1:** Demographic and clinical characteristics of patient cohorts.

	HCV	NAFLD	Healthy controls
No. of patients; n	39	34	11
Age, years; median (range)	57.9 (32.7–67.4)	60.4 (27.4–79.2)	55.5 (24–84)
Gender, male; n (%)	25 (64.1)	19 (55.9)	6 (54.5)
BMI, kg/m^2^; median (range)	26.7 (18–42.6)	31.4 (22.8–53.5)	
**Cirrhosis status; n (%)**			
No advanced fibrosis	16 (41)	11 (32.3)	-
Cirrhotic	12 (30.8)	14 (41.2)	-
Decompensated	11 (28.2)	9 (26.5)	-
**Viral Genotype; n (%)**			
1 a/b	25 (64.1)	-	-
2	2 (5.1)	-	-
3	12 (30.8)	-	-

### NAFLD is associated with an increase in peripheral monocyte CD16^+^ subset regardless of disease stage

**Fig 1:**
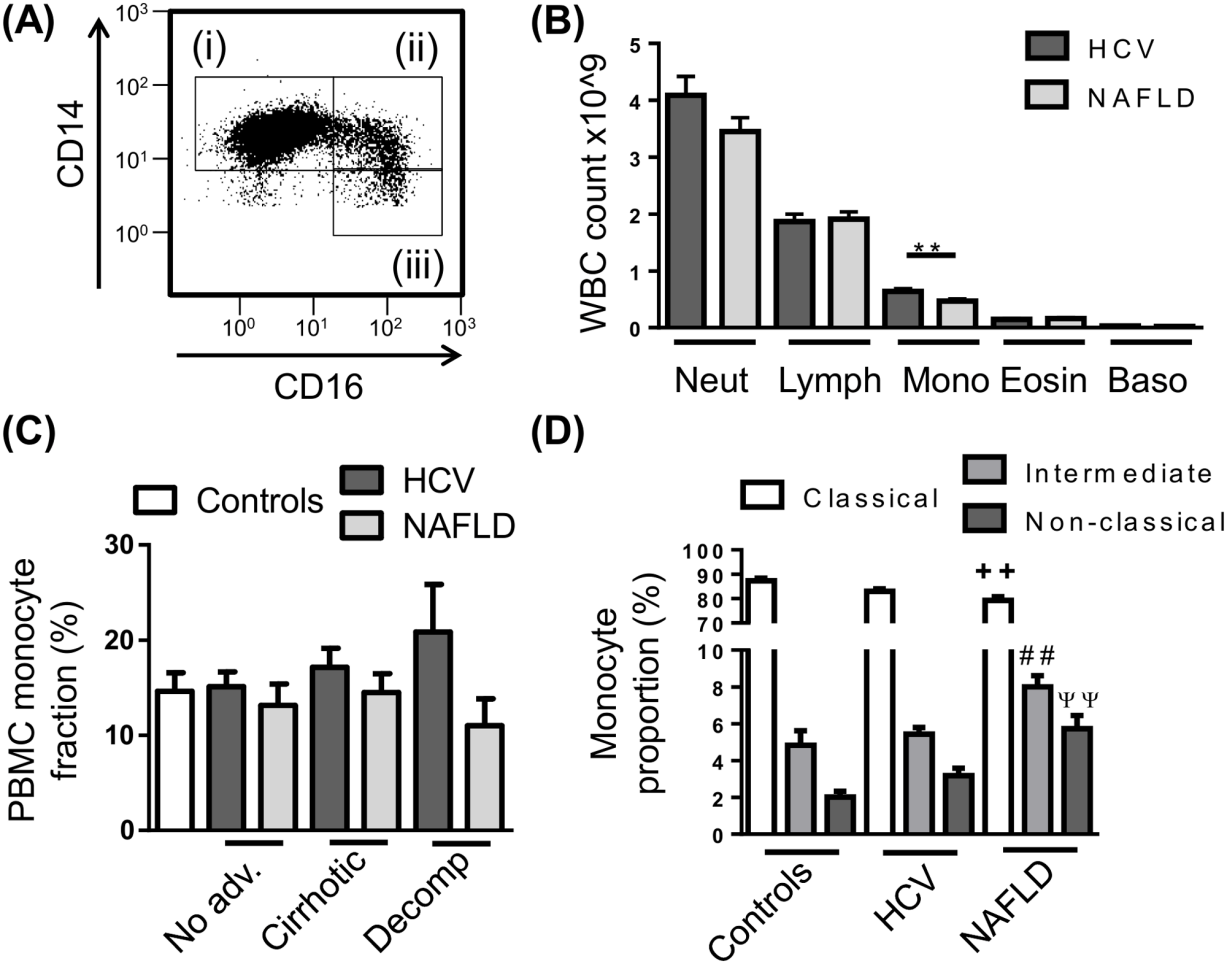
Peripheral blood monocyte subset distribution in chronic liver disease (CLD). (A) Representative flow cytometry plot of the 3 main monocyte subsets, i—CD14^high^CD16^-^ “Classical”, ii—CD14^high^CD16^+^ “intermediate”, iii—CD14^+^CD16^+^ “non-classical” monocytes. (B) Distribution of peripheral leukocyte lineages obtained from patient haematology full blood counts. (C) Proportion of monocytes in isolated peripheral blood mononuclear cell (PBMC) in control subjects and CLD patients with no-advanced fibrosis (No adv.), cirrhosis or decompensated cirrhosis (Decomp). (D) Distribution of monocyte subsets in the PBMC fraction of control subjects and CLD patients. (Data represented as mean +SEM, ** ^++ ## ψψ^ P<0.01; D, significance shown vs corresponding control subset; Neut, neutrophils; Lymph, lymphocytes; Mono, monocytes; Eosin, eosinophils; Baso, basophils)

To determine whether circulating monocyte subsets are altered in different etiologies or stages of disease, PBMC were isolated from control subjects and patients with HCV or NAFLD, stained for monocyte markers CD14 and CD16 and assessed by flow cytometry (Fig 1A). Automated clinical complete blood cell counts revealed modestly elevated numbers of monocytes in patients with HCV compared to NAFLD (Fig 1B), however this was not observed following PBMC isolation, where the proportion of monocytes within the PBMC fraction was comparable between control subjects and CLD patients regardless of etiology and stage of disease (Fig 1C). In our cohort of patients with NAFLD, monocyte subsets were skewed towards the CD16^+^ populations with an increase in both intermediate and non-classical subsets and a commensurate decrease in classical monocytes compared to control subjects, however this was not seen in patients with HCV (Fig 1D). This increase in the CD16^+^ monocyte subsets was not attributed to a specific stage of disease (data not shown). The proportion of monocytes (total or subsets) was not associated with clinical or demographic parameters, including age, gender, BMI, ALT/AST levels, MELD score, fibroscan result, neutrophil to lymphocyte ratio or viral genotype.

### Reduced monocyte CCR2 expression in HCV and NAFLD

**Fig 2:**
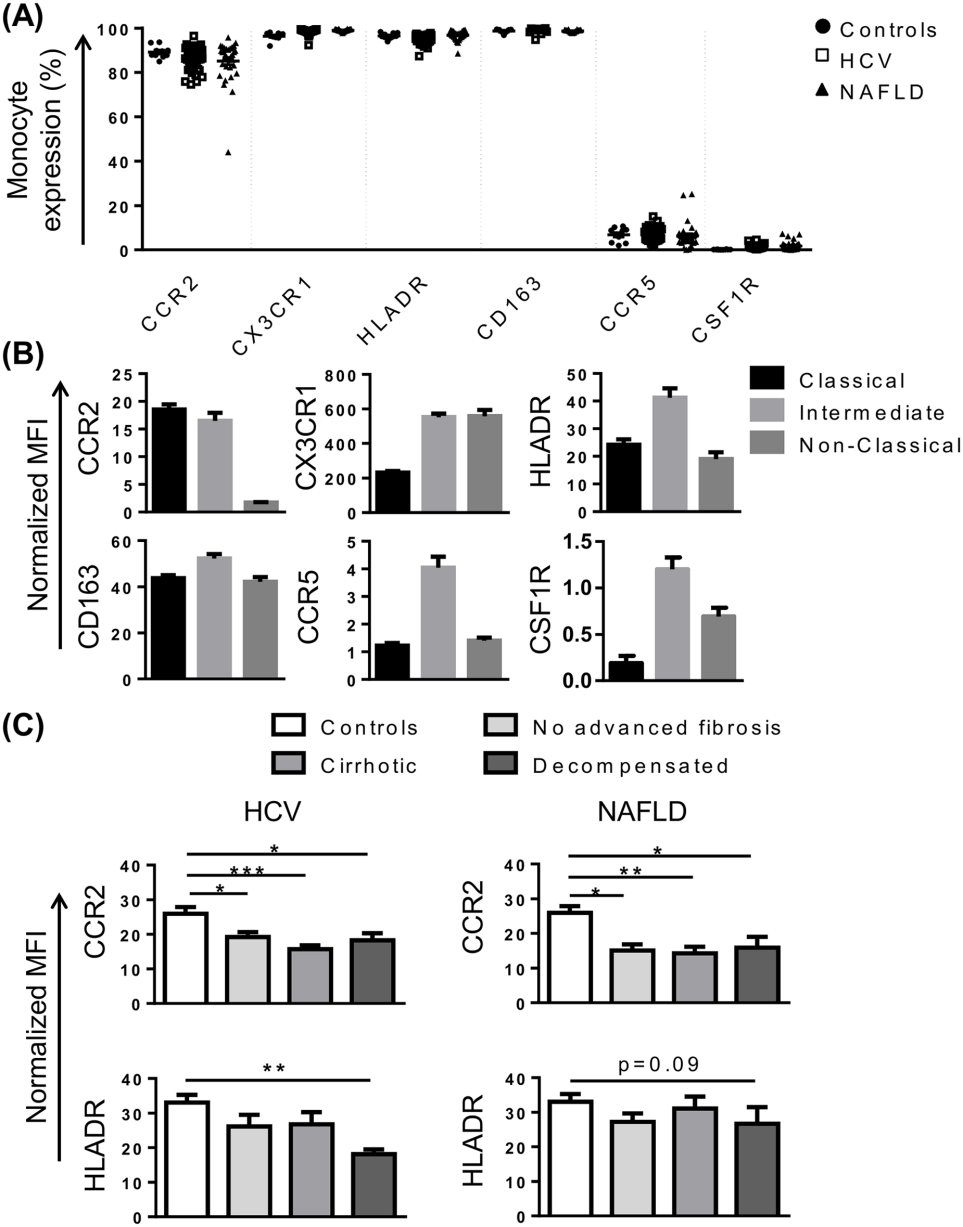
Characterisation of monocyte phenotype in chronic liver disease (CLD). (A) Proportion of monocytes positive for common subset markers in control subjects and patients with HCV or NAFLD. (B) Level of expression (median fluorescence intensity, MFI) of selected markers on the 3 main monocyte subsets in patients with HCV. Figures are representative of subset expression in patient and control cohorts. (C) Monocyte CCR2 and HLA-DR expression in control subjects and CLD patients with no advanced fibrosis, cirrhosis or decompensated cirrhosis. (Data represented as mean +SEM, * P<0.05, **P<0.01, **P<0.001)

In addition to CD14 and CD16, phenotypic differences between monocyte subsets have been identified, and related to differences in function [9]. The distribution of expression of our selected phenotypic markers differed between the three monocyte subsets, and this distribution was conserved across both patient and healthy cohorts. As expected, monocytes were positive for the established subset markers CCR2 and CX3CR1 with preferential expression on CD14^high^/CD16^-^ classical and CD14^+^/CD16^+^ non-classical monocytes respectively, in both control subjects and CLD patients (Fig 2A and 2B). Similarly, HLA-DR, CD163, CCR5 and colony stimulating factor receptor (CSF1R) were highly expressed on CD14^high^/CD16^+^ intermediate monocytes (Fig 2B), although CCR5 and CSF1R were expressed at very low levels (Fig 2A). Interestingly, in both HCV and NAFLD, monocyte CCR2 expression was significantly lower when compared to healthy controls (Fig 2C).

### Reduced monocyte HLA-DR expression in decompensated cirrhosis

Previous studies have suggested patients with advanced cirrhosis display an “immune paralysis” phenotype, characterised by low HLA-DR expression, contributing to increased mortality [26, 27]. In our cohort, HLA-DR expression was significantly reduced in patients with HCV-related decompensated cirrhosis (P<0.01), and similarly reduced in patients with NAFLD, although this did not reach statistical significance (P = 0.09) (Fig 2C). Elevated levels of soluble and monocyte CD163 expression have also been reported in acute-on-chronic liver failure [28], and may be predictive of hospital patient mortality in patients with sepsis [29]. CD163 levels were similar in patients with NAFLD and control subjects, but were positively correlated with MELD score in patients with HCV (r = 0.53, P = 0.001).

### Altered monocyte expression of CCR4, CXCR3, CXCR4 and CD62L in CLD

**Fig 3:**
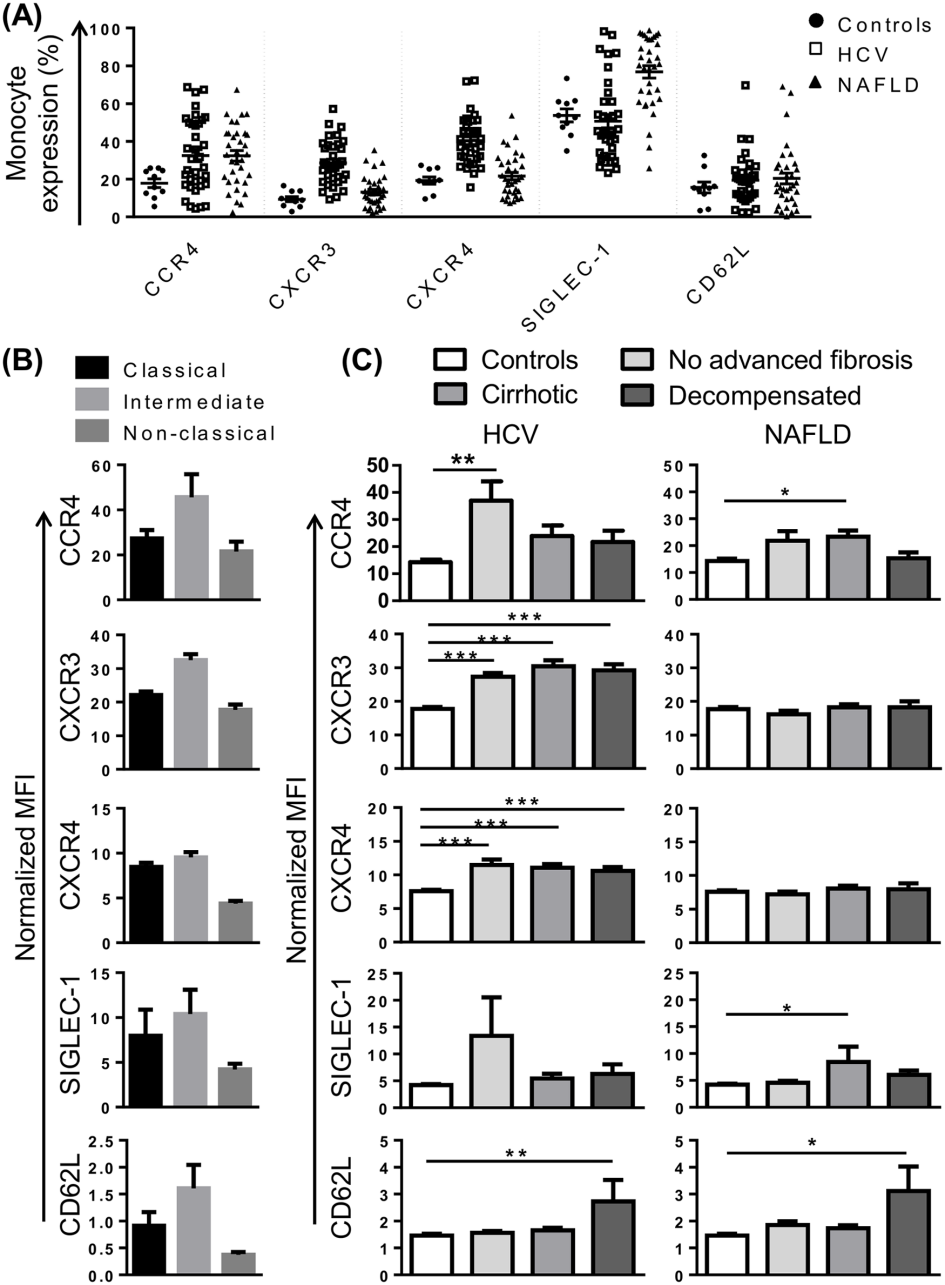
Alterations in chemokine receptor expression and adhesion molecules implicated in monocyte recruitment in patients with chronic liver disease (CLD). (A) Proportion of monocytes positive for selected chemokine receptors and adhesion molecules in control subjects and patients with HCV or NAFLD. (B) Level of expression (median fluorescence intensity, MFI) of selected markers on the 3 main monocyte subsets in patients with HCV. Figures are representative of subset expression in patient and control cohorts. (C) Expression of selected markers on monocytes from control subjects and CLD patients with no advanced fibrosis, cirrhosis or decompensated cirrhosis. (Data represented as mean +SEM, *P<0.05, **P<0.01, ***P<0.001)

To gain further insight into monocyte phenotype in CLD, we selected a panel of markers that, in addition to CX3CR1 and CCR2, may be involved in monocyte recruitment to the injured liver. CCR4, a chemokine receptor that was over-expressed in portal areas of patients with HCV, may recognise several chemokines identified in portal tracts or otherwise implicated in CLD progression, including CCL4, CCL5, CCL2 [15]. In our cohort, CCR4 was expressed on a variable proportion of monocytes (2–67%), most commonly on intermediate monocytes and a subset of classical monocytes (Fig 3A and 3B). CCR4 expression was significantly increased in patients with CLD compared to controls, regardless of disease stage or etiology (Fig 3C). Chemokine (C-X-C motif) receptor (CXCR)3 is the receptor for chemokines CXCL9-11 and CXCL4 that are upregulated in HCV infected liver and/or peripheral blood [15, 24, 25], and liver CXCR3 expression correlated with portal T-cell accumulation [15]. CXCR3 was most highly expressed on intermediate monocytes (Fig 3B), and significantly increased in patients with HCV, but not NAFLD, regardless of disease stage (Fig 3C). CXCR4, the receptor for CXCL12, a chemokine expressed by inflamed biliary epithelium in primary biliary cirrhosis and HCV [16], was enriched in portal compared to parenchymal regions in HCV patients with advanced fibrosis [24]. CXCR4 was highly expressed on CD16^-^ monocyte subsets (Fig 3B) and, like CXCR3, increased in patients with HCV but not NAFLD, regardless of disease stage (Fig 3C).

In addition to chemokine receptors, we investigated the expression of the selectin CD62L and sialic acid binding Ig-like lectin (SIGLEC)-1 (also known as CD169) as monocyte recruitment is also highly dependent on adhesion molecules. SIGLEC-1 is specifically expressed on myeloid cells [30], and was among the most highly upregulated genes in HCV PBMC at the mRNA level [25]. In our cohort, CD14^high^ monocytes displayed higher SIGLEC-1 expression than the CD14^+^CD16^+^ subset (Fig 3B). There was a modest increase in SIGLEC-1 expression in cirrhotic patients with NAFLD compared to controls, but no alterations in patients with HCV (Fig 3C). CD62L has been shown to be more highly expressed on inflammatory liver macrophages compared to restorative macrophages in mouse models of CLD [3] and the corresponding human CD14^high^CD16^-^ subset also displays high levels of gene expression [31]. In our cohort, CD62L was most highly expressed on intermediate monocytes (Fig 3B), however a small proportion of CD62L^+^ monocytes was observed within each subset suggesting that CD14^+^CD62L^+^ monocytes may represent a unique population, masked by current gating strategies. Monocyte CD62L expression increased in HCV and NAFLD patients with decompensated cirrhosis (Fig 3C). CD62L expression was positively correlated with MELD score in both HCV and NAFLD cohorts (r = 0.51 P = 0.002 and r = 0.4 P = 0.02, respectively).

### Monocytes from patients with CLD have reduced functional capacity

Monocytes have a crucial role in extracellular bacterial and fungal clearance through phagocytosis and oxidative responses. Impaired monocyte function may contribute to susceptibility to infection observed in end-stage liver disease or prevent effective immune responses during early stages of disease. Here, phagocytic capacity, oxidative responses and inflammatory cytokine production were assessed to determine the consequences of persistent hepatic damage due to viral or metabolic injury.

**Fig 4:**
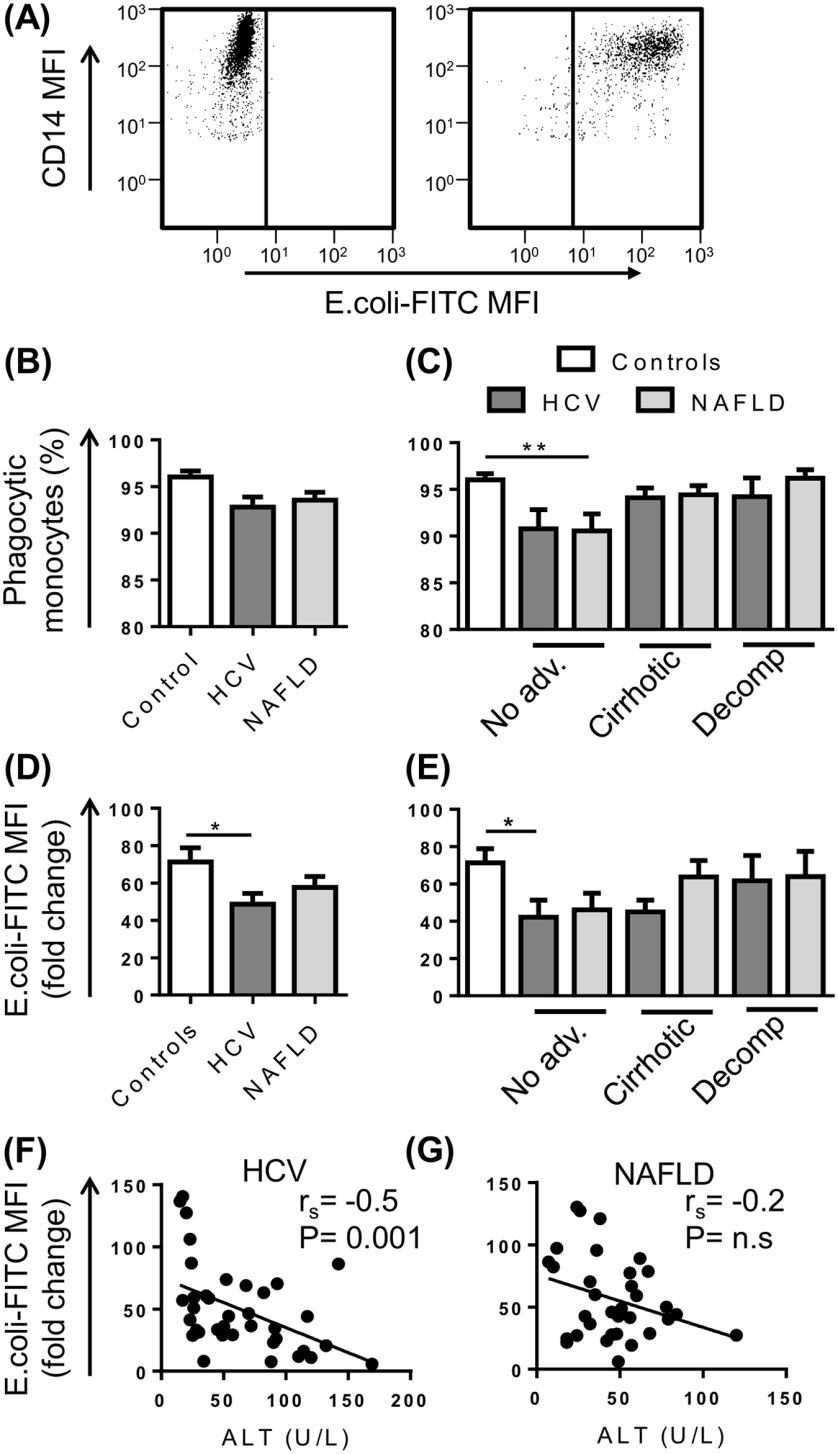
Phagocytic capacity of peripheral blood monocytes in patients with chronic liver disease (CLD). (A) Representative flow cytometry plots highlighting CD14^+^ monocyte uptake of E.Coli FITC-labelled BioParticles. The proportion of monocytes undergoing phagocytosis (B and C) and monocyte phagocytic capacity (D and E) in control subjects and CLD patients with no advanced fibrosis (No adv.), cirrhosis or decompensated cirrhosis (Decomp). (F and G) Correlation between monocyte phagocytic capacity and patient serum ALT levels. (Data represented as mean +SEM, * P<0.05, **P<0.01)

Monocyte phagocytic capacity for fluorescently labelled *E*.*coli* BioParticles was assessed by flow cytometry (Fig 4A). Greater than 90% of monocytes from control subjects and patients with CLD phagocytosed FITC-labelled *E*.*coli* particles (Fig 4B and 4C), with CD14^high^ monocytes having the largest phagocytic capacity (increased fluorescence intensity)(data not shown). Monocytes from patients with HCV had a reduced phagocytic capacity compared to control subjects (P<0.05) (Fig 4D), which was more prominent prior to decompensation (Fig 4E). Monocytes from patients with NAFLD showed a similar trend, but this did not reach statistical significance (P = 0.2) (Fig 4D and 4E). Monocyte phagocytic capacity was inversely correlated with increasing ALT levels (r_s_ = -0.5061 P<0.01) (Fig 4F) but not AST levels (r_s_ = -0.2763, P = 0.09) in patients with HCV, but not NAFLD (Fig 4G).

**Fig 5:**
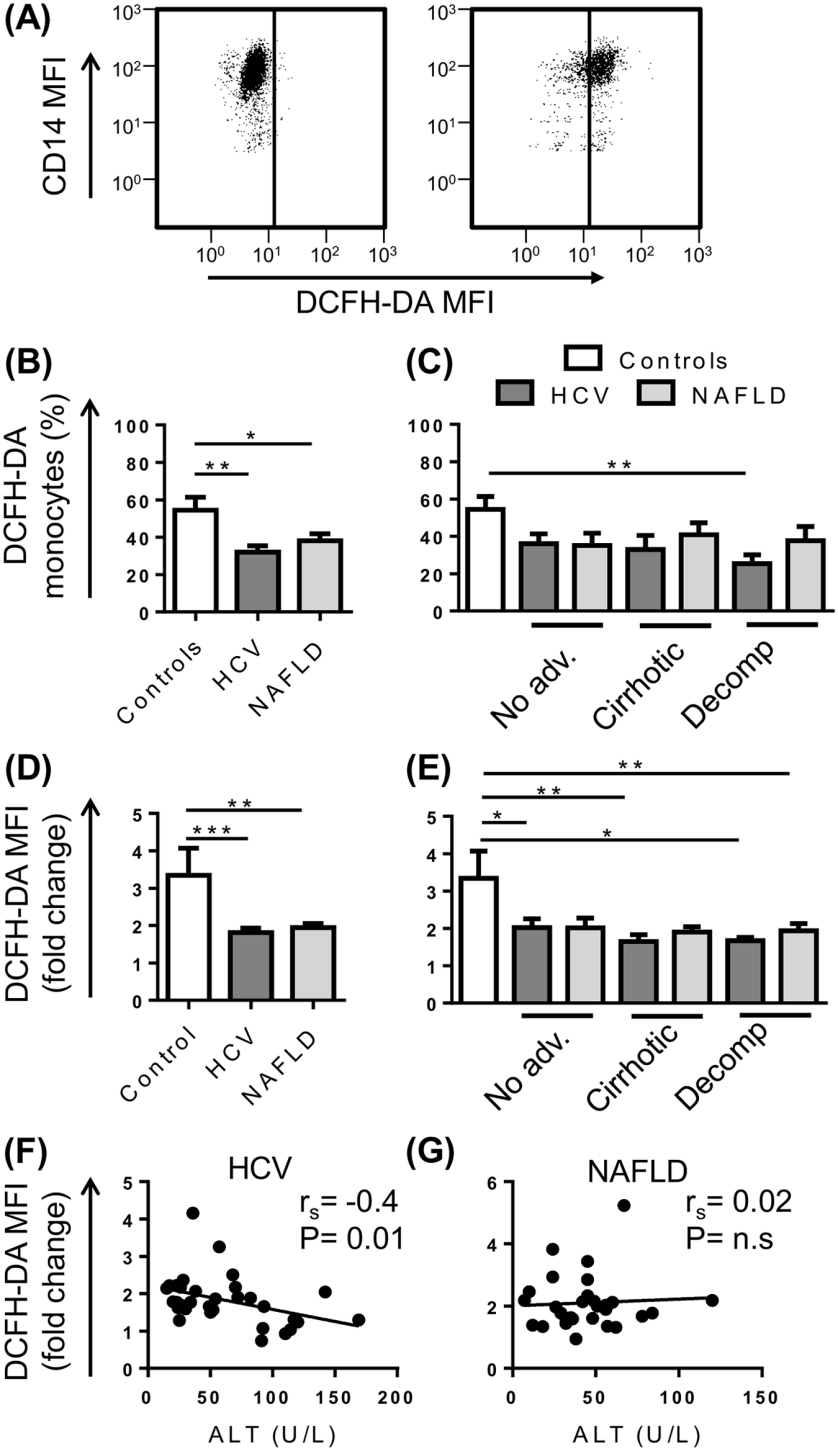
Oxidative burst capacity of peripheral blood monocytes in patients with chronic liver disease (CLD). (A) Representative flow cytometry plots highlighting CD14^+^ monocyte generation of free oxygen radicals at baseline or upon stimulation with opsonised zymosan. The proportion of monocytes capable of generating reactive oxygen species (B and C) and monocyte capacity to produce ROS (D and E) following stimulation with opsonised zymosan in control subjects and CLD patients with no advanced fibrosis (No adv.), cirrhosis or decompensated cirrhosis (Decomp). (F and G) Correlation between monocyte oxidative burst capacity and patient serum levels. (Data represented as mean +SEM, * P<0.05, **P<0.01, ***P<0.001; DCFH-DA, dichlorodihydrofluorescein).

Monocyte capacity to generate a microbicidal oxidative burst was assessed by their production of ROS in response to serum-opsonised zymosan using the oxidisable fluorescent probe DCFH-DA (Fig 5A). CD14^high^ and CD14^low^ monocytes had equal capacity to produce ROS. Fewer monocytes from both HCV (P<0.01) and NAFLD (P<0.05) patient cohorts produced ROS upon stimulation when compared to control subjects (Fig 5B), which was consistent across all stages of disease but only reached statistical significance for HCV patients with decompensated cirrhosis (Fig 5C). The amount of ROS generated by monocytes was also significantly blunted (P<0.01) (Fig 5D), regardless of disease stage (Fig 5E). In addition, similar to phagocytic capacity, ROS generation by monocytes from patients with HCV, but not NAFLD, inversely correlated with ALT levels (r_s_ = -0.4268 P<0.5) (Fig 5F and 5G).

**Fig 6:**
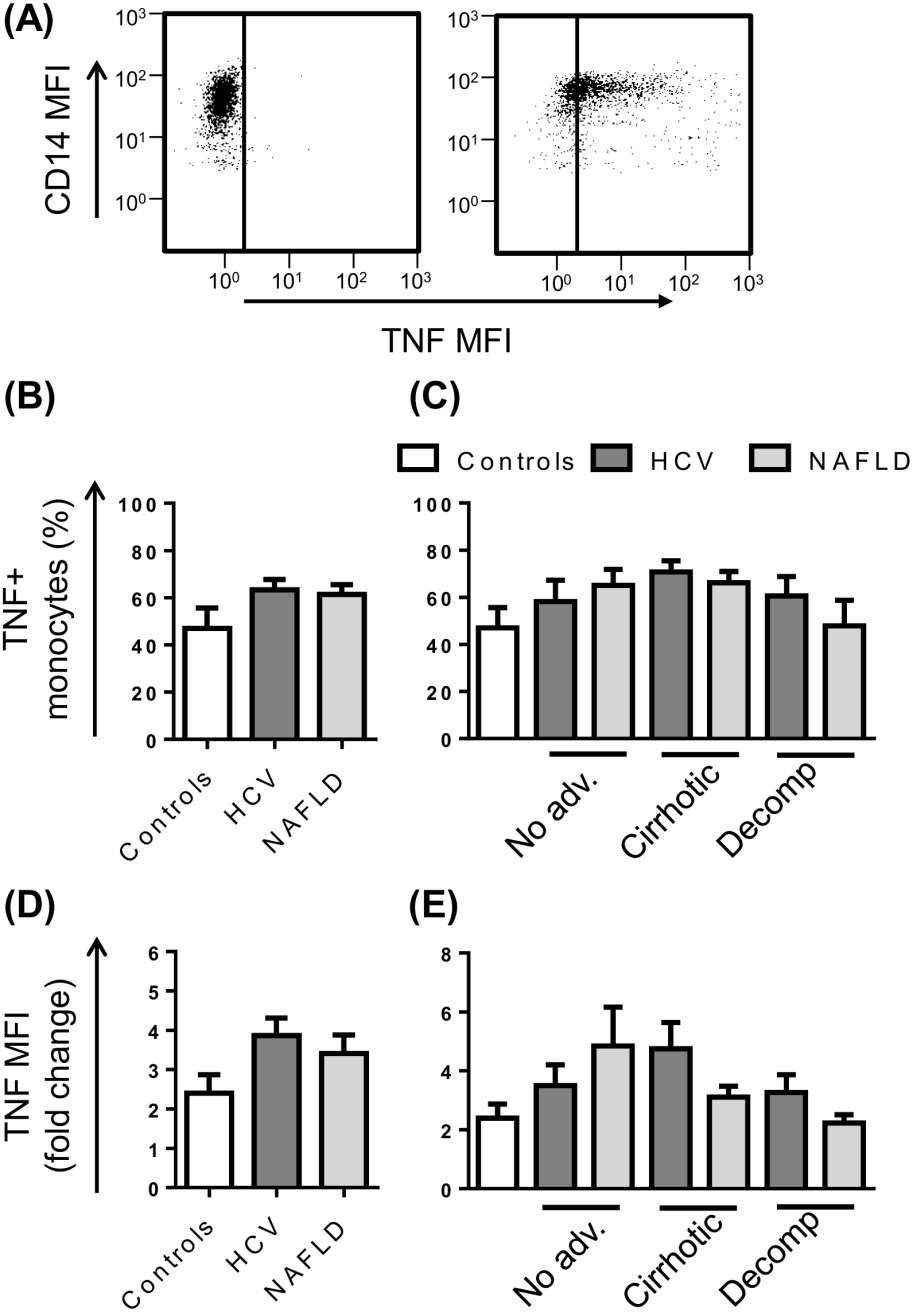
Monocyte TNF production in patients with chronic liver disease (CLD). (A) Representative flow cytometry plots highlighting monocyte specific TNF production at baseline or upon stimulation with LPS. The proportion of monocytes producing TNF (B and C) and the level of TNF production (D and E) following stimulation with LPS in control subjects and CLD patients with no advanced fibrosis (No adv.), cirrhosis or decompensated cirrhosis (Decomp). (Data represented as mean +SEM).

Inflammatory cytokine production was assessed by intracellular staining for the prototypical cytokine, TNF, after 4 hours *ex vivo* stimulation with LPS in the presence of the protein transport inhibitor Brefeldin A (Fig 6A). In the absence of activating stimuli, monocytes were largely negative for TNF, with the exception of 3 patients who displayed production in >50% of cells. Upon stimulation with LPS, the CD14^high^ and CD14^low^ subsets were equally capable of producing TNF and the percentage of monocytes that responded ranged from 8–95%. The proportion of TNF producing monocytes and the level of cytokine produced upon stimulation (fluorescence intensity) did not differ between control subjects and patients irrespective of stage of disease (Fig 6B and 6E).

### Reduced Monocyte TNF production is associated with early mortality in patients with decompensated cirrhosis

**Fig 7:**
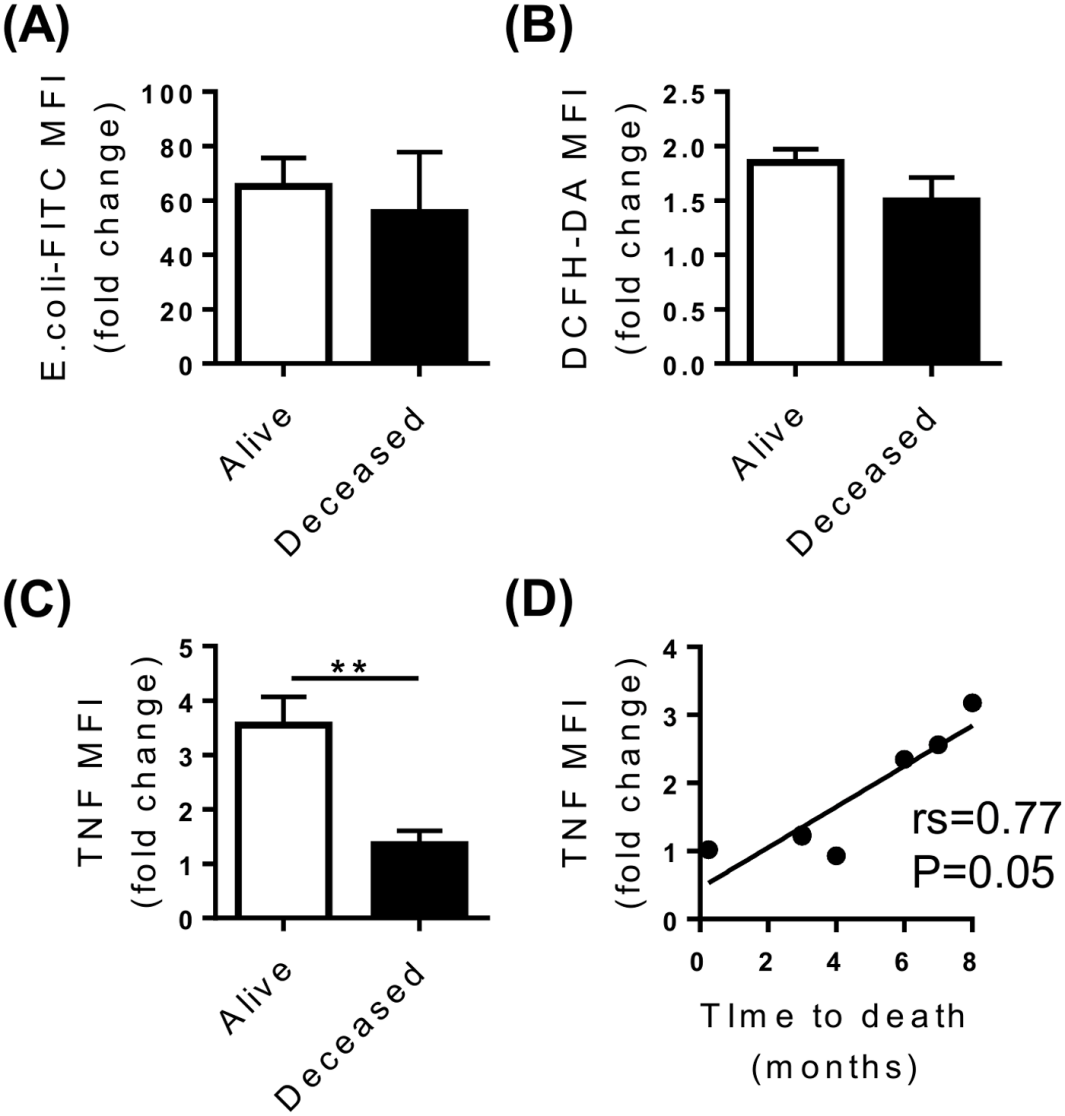
Monocyte function and 6 month mortality. Patient survival and monocyte phagocytic capacity (A), zymosan-stimulated oxidative burst (B) and LPS-stimulated TNF production (C) in combined decompensated HCV and NAFLD cohorts. (D) Correlation between monocyte LPS-stimulated TNF production and time to death. (Data represented as mean +SEM, ** P<0.01; DCFH-DA, dichlorodihydrofluorescein).

The development of life-threatening infections is a common feature of decompensated cirrhosis and is associated with significant patient morbidity and mortality. Having observed features of monocyte dysfunction in our cohort, we assessed whether monocyte dysfunction was indicative of 6 month mortality specifically in patients with decompensated cirrhosis. Five patients with decompensated cirrhosis died within 6 months of recruitment, and a further 3 were deceased by 8 months. Monocyte phagocytic capacity and oxidative responses were not associated with 6 month mortality (Fig 7A and 7B), however monocyte TNF production in response to LPS was significantly blunted in patients who died within 6 months of inclusion in the study (P = 0.01) (Fig 7C). Furthermore, monocyte TNF production positively correlated with time to death (r_s_ = 0.773, P = 0.05) (Fig 7D) suggesting that dysregulated cytokine production may contribute to mortality in these patients.

## Discussion

Peripheral blood monocytes are recruited to the chronically injured liver where they have a key role in driving hepatic inflammation and fibrogenesis. Despite their contribution to disease processes at the *site* of injury, whether *circulating* monocyte subsets are altered and how they are recruited in liver disease of diverse etiologies or at different stages of disease remains poorly understood.

Our data show that monocyte subsets are skewed towards the CD16^+^ populations, irrespective of the stage of liver disease or clinico-demographic factors, in patients with NAFLD but not HCV. A similarly modest increase in CD16^+^ monocytes was previously observed in a very large, mixed etiology CLD cohort [18], in hepatitis B [11] and NAFLD [32]. Other studies, however, failed to find statistically significant differences in peripheral blood monocyte subsets between CLD patients and healthy controls [8, 17], underscoring the inter-individual variability in monocyte populations and relatively small effect size. Increased frequency of CD16^+^ monocytes has also been demonstrated in obesity, type 2 diabetes, cardiovascular disease and dyslipidemia, metabolic conditions commonly seen in association with NAFLD [33–35]. Although these studies support a connection between adiposity, inflammation and CD16^+^ monocytes, it remains unclear whether the alteration in monocyte subsets is a consequence of, or directly adds to the inflammatory response. Of concern, CD14^high^CD16^+^ monocyte counts independently predicted cardiovascular events in a large patient cohort referred for elective coronary angiography (n = 951)[36] and another patient cohort with chronic kidney disease (n = 438)[37]. Longitudinal clinical outcome studies in people with NAFLD will be important to determine whether the frequency of CD16^+^ monocytes is a predictive biomarker for cardiovascular disease.

In contrast to NAFLD, HCV patients had a different monocyte phenotypic signature, with increased expression of CXCR3 and CXCR4 regardless of disease stage. Multiple CXCR3 ligands, including CXCL9, CXCL10 and CXCL11, are products of interferon-stimulated genes, which may suggest this phenotype is a response to chronic viral infection. Elevated hepatic CXCR3 expression, and peripheral CXCR3 chemokine levels, have been associated with liver inflammation and fibrosis in chronic HCV [15, 38]. Although studied in the context of T cell recruitment to inflamed peripheral tissues, including the liver [39, 40], the role of CXCR3 in hepatic monocyte recruitment, or activation, is unknown. The CXCL12/CXCR4 axis is also implicated in lymphocyte recruitment to the liver in chronic HCV, with infiltrating T and natural killer cells expressing high levels of CXCR4 [41]. However, despite known roles in tissue injury [42], the CXCL12/CXCR4 axis may have a *protective* role during inflammation and injury by promoting liver regeneration and repair or controlling mobilisation of inflammatory cells from the bone marrow [43]. CXCL12, and the CXCR3 ligands CXCL9-11, are expressed in inflamed portal tracts [15, 16], suggesting the hypothesis, that these axes contribute to monocyte recruitment specifically to the portal niche, which contains an abundance of macrophages, myofibroblasts and activated hepatic progenitor cells that are associated with CLD progression [6, 15]. Further studies to assess the expression of CXCR3 and CXCR4 on intrahepatic monocytes, and their location, will provide further evidence as to the role of these chemokine axes in liver inflammation and fibrogenesis.

In both chronic HCV and NAFLD, monocyte CCR2 expression was significantly lower (confirming a previous study [18]) and CCR4 expression was significantly higher compared to control subjects, regardless of disease stage. Although high levels of the CCR2 ligand, CCL2, are observed in the serum and liver of patients [13, 14], the contribution of this axis to human CLD is unclear. The reduction in CCR2 expression may reflect a feedback mechanism to control monocyte mobilisation and tissue recruitment, in response to the increased ligand or inflammatory stimuli. This finding corroborates our previous data demonstrating a reduction in the number of CCR2^+^ liver macrophages in patients with HCV [7]; and further implicates the CCR2 axis in human CLD. In mice, genetic deletion or pharmacological inhibition of CCR2 impaired monocyte infiltration and modestly reduced fibrogenic parameters in a number of hepatic injury models, although injury often remained significantly elevated over controls [5, 44]. However CCR2, but not CCL2, was dispensable for thioacetamide-induced monocyte recruitment and subsequent liver injury [2], implicating additional CCL2 receptors, such as CCR4 [45], which was elevated in our patient cohort, in hepatic monocyte recruitment. In contrast to CCR2 and CCR4, CD62L, a selectin implicated in preferential recruitment of blood monocytes to sites of inflammation [46], was specifically increased on monocytes from patients with decompensated cirrhosis. CD62L expression positively correlated with MELD score, which may suggest increased hepatic recruitment of inflammatory monocytes exacerbates tissue injury in these patients.

In addition to providing insight into hepatic recruitment, circulating monocyte phenotype may be a valuable indicator of the systemic inflammatory state and monocyte immunocompetence. Monocytes are critical mediators of host defence, and infections are responsible for much of the morbidity, mortality and resource utilisation in patients with decompensated cirrhosis. We demonstrate that monocytes from CLD patients have reduced capacity to produce ROS in response to zymosan stimulation, regardless of etiology or disease stage. These data suggest impaired antimicrobial function in the setting of CLD, although increased susceptibility to infection, which is only clinically manifest in patients with decompensated cirrhosis, is undoubtedly multi-factorial.

In line with previous studies [27], we confirmed reduced monocyte HLA-DR expression in decompensated cirrhosis which would likely impair antigen presentation capacity and adaptive immune responses. Reduced monocyte HLA-DR levels and inability to recover expression predicted poor prognosis and paralleled liver disease severity [23], leading to significant interest in the therapeutic potential of agents that can restore monocyte HLA-DR expression [47]. Reduced monocyte HLA-DR expression and capacity for LPS-stimulated TNF induction, which was associated with early mortality in our cohort, are hallmarks of innate immune hyporesponsiveness, or ‘immunoparalysis’, that occurs in septic and other critically ill patients, rendering them susceptible to infections. In cirrhosis, and particularly decompensated cirrhosis, this immunosuppressed state may result from continuous translocation of gut microbes and microbial products due to increased intestinal permeability, which can cause infections, but also increase portal pressure, impair liver function and worsen haemostasis [48, 49]. Whether reduced TNF production is simply a biomarker of rapid decline or contributed directly to the death of these patients, in the context of clinical or sub-clinical infections or other pathological processes, is not known. The association between TNF production and mortality, but not decompensated cirrhosis per se, might help us to make sense of apparently contradictory reports of increased [50, 51] as well as reduced [26, 27] monocyte TNF production in cirrhosis. Serum TNF has also been associated with cirrhosis severity, contributing to endothelial activation and hemodynamic disturbance [50]. Although we did not measure serum TNF in this study it has been suggested, paradoxically, that reduced stimulated TNF production can co-exist with high serum TNF levels [52].

Although the persistence of inflammatory macrophages is associated with liver fibrosis, anti-fibrotic roles for macrophages have also been demonstrated in mouse models, both during active fibrogenesis and disease resolution [53, 54]. Macrophage cell therapy is an area of active investigation, as delivery of mature mouse macrophages (but not their precursors) to mice with toxin-induced chronic liver disease ameliorated fibrosis by dampening inflammation and promoting matrix degradation [55]. Injection of human monocyte-derived macrophages in a murine fibrosis model similarly led to a reduction in markers of liver injury and fibrosis, and an increase in markers of liver regeneration [56]. More recently, the feasibility of using autologous monocyte-derived macrophages as therapy for human CLD was tested, where circulating monocytes from patients with cirrhosis and healthy control subjects were shown to differentiate into functionally comparable macrophages [56]. Whether or not monocytes from patients with decompensated cirrhosis, that display features of monocyte dysfunction, could be restored *in vitro* into functional macrophages may have important implications for cell-based therapy.

In summary, the current study highlights distinct differences in monocyte phenotype and function in patients with chronic HCV and NAFLD and provides insight into how specific injury-induced signals from the liver may alter peripheral blood monocyte phenotype. The relationship between circulating monocyte phenotype and intrahepatic monocyte/macrophage phenotype, localisation and function is not clear; it is not known whether distinct monocyte subsets are recruited to fulfil site-specific functions in CLD or whether circulating monocytes are recruited indiscriminately and alter their phenotype and function in response to the injured microenvironment. These are important questions to answer, as altering the recruitment of specific etiology-dependent monocyte subsets or delivering specific monocyte/macrophage populations are appealing targets for anti-fibrotic therapies in patients with CLD. Furthermore, understanding how the balance between monocyte immunosuppressive and immunoprotective phenotypes effects patient clinical outcomes may aid in the management of end-stage liver disease and provide novel therapeutic targets to restore monocyte function to reduce susceptibility to infection.

## Supporting Information

S1 Fig Representative histograms of phenotypic marker expression on classical CD14+CD16- and non-classical CD14+CD16- on peripheral blood monocyte subsets. Monocytes were gated based on forward/side scatter properties and CD14/CD16 expression. Median fluorescence intensities (MFI) of selected phenotypic markers were normalized by subtracting the MFI of the corresponding fluorescence-minus-one control channel.
(TIF)

## Abbreviations

CLD: chronic liver disease
HCV: chronic hepatitis C
NAFLD: non-alcoholic fatty liver disease
CCR: chemokine (C-C motif) receptor
CX3CR: chemokine (C-X3-C motif) receptor
CCL: chemokine (C-C motif) ligand
ROS: reactive oxygen species
HLA-DR: major histocompatibility complex class II surface receptor
PBMC: peripheral blood mononuclear cells
DCFH-DA: dichlorodihydrofluorescein
TNF: tumour necrosis factor
LPS: lipopolysaccharide
MFI: median fluorescence intensity
MELD: model for end stage liver disease
CSF1R: colony stimulating factor receptor
CXCR: chemokine (C-X-C motif) receptor
SIGLEC: sialic acid binding Ig-like lectin
