# Supplementary figures and images for: Altered Peripheral Blood Monocyte Phenotype and Function in Chronic Liver Disease: Implications for Hepatic Recruitment and Systemic Inflammation
Source: PLoS One. 2016 Jun 16;11(6):e0157771. doi: 10.1371/journal.pone.0157771 (PMC4911107; doi:10.1371/journal.pone.0157771)

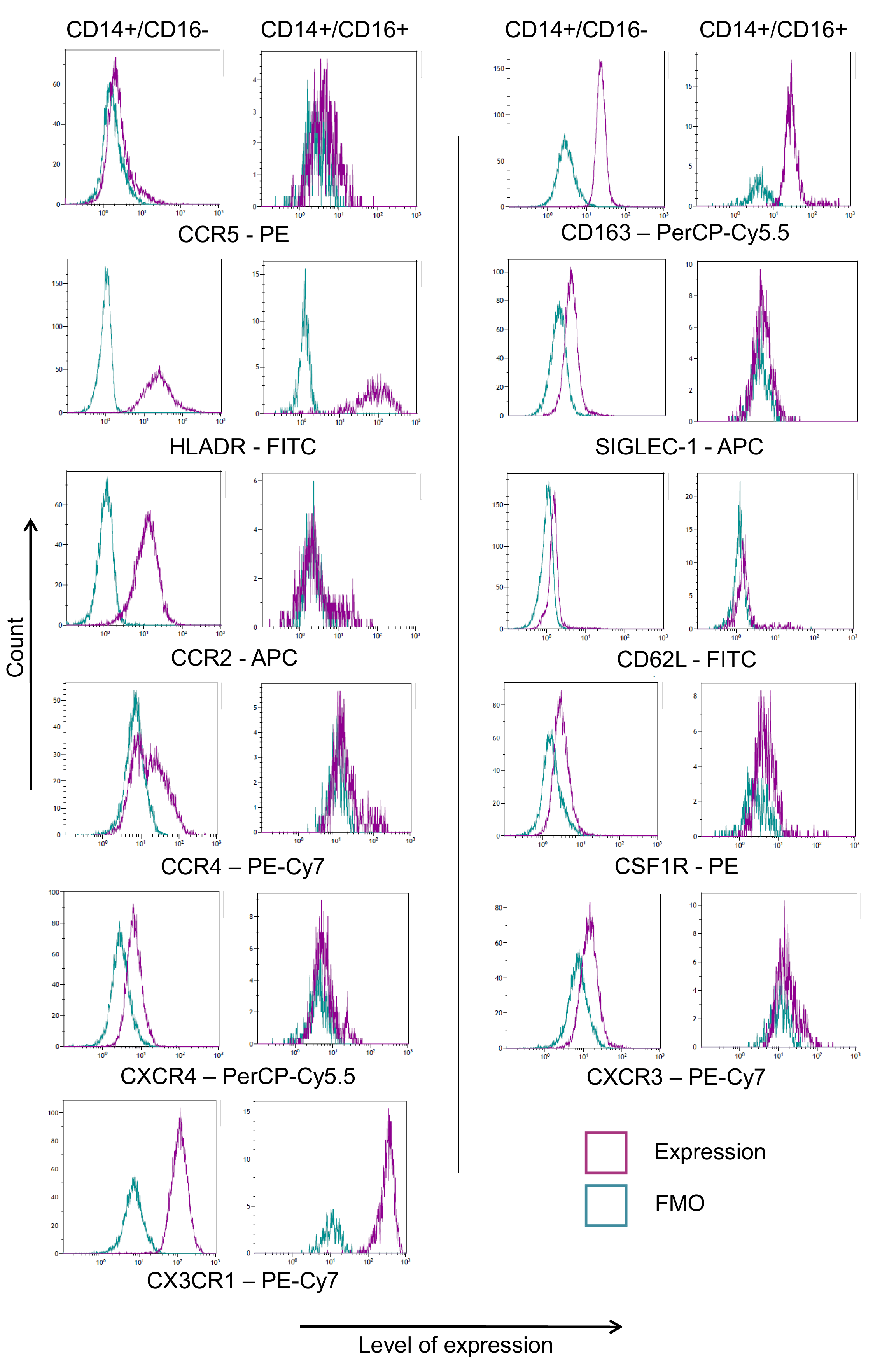

Supplement: S1 Fig — Monocytes were gated based on forward/side scatter properties and CD14/CD16 expression. Median fluorescence intensities (MFI) of selected phenotypic markers were normalized by subtracting the MFI of the corresponding fluorescence-minus-one control channel. (TIF) [file pone.0157771.s001.tif]
